# Comparing Cognitive Profiles of Licensed Drivers with Mild Alzheimer's Disease and Mild Dementia with Lewy Bodies 

**DOI:** 10.1155/2016/6542962

**Published:** 2016-09-27

**Authors:** Stephanie Yamin, Arne Stinchcombe, Sylvain Gagnon

**Affiliations:** ^1^School of Psychology, University of Ottawa, Ottawa, ON, Canada; ^2^Faculty of Human Sciences, Saint Paul University, Ottawa, ON, Canada

## Abstract

*Purpose*. Alzheimer's disease (AD) and dementia with Lewy Bodies (DLB) constitute two of the most common forms of dementia in North America. Driving is a primary means of mobility among older adults and the risk of dementia increases with advanced age. The purpose of this paper is to describe the cognitive profile of licensed drivers with mild AD and mild DLB.* Method*. Licensed drivers with mild AD, mild DLB, and healthy controls completed neuropsychological tests measuring general cognition, attention, visuospatial/perception, language, and cognitive fluctuations.* Results*. The results showed differences between healthy controls and demented participants on almost all neuropsychological measures. Participants with early DLB were found to perform significantly worse on some measures of attention and visuospatial functioning in comparison with early AD.* Discussion*. Future research should examine the relationship between neuropsychological measures and driving outcomes among individuals with mild AD and mild DLB.

## 1. Introduction

The Canadian population is aging, a trend that is expected to continue for the next several decades due to a reduced fertility rate, an increase in life expectancy, and the aging of the baby boom cohort. In 2011, an estimated 5 million Canadians were 65 years of age or older, a number that is expected to double in the next 25 years to reach approximately 10.4 million older adults by 2036 [1]. Aging is associated with an increased risk of chronic illness including cardiovascular disease, cancer, arthritis, cataracts, osteoporosis, type 2 diabetes, hypertension, and dementia [2]. In addition, while it is well-documented that aging is associated with senescent cognitive changes, some older adults will experience more significant cognitive impairment related to the aforementioned conditions.

The most common type of age-related cognitive impairment is dementia. Dementia is an umbrella term for a syndrome that includes cognitive impairment in several domains (e.g., memory, attention, language, executive function, and visuospatial abilities) and functional impairment as measured through the assessment of activities of daily living (ADL; i.e., eating, bathing, dressing, toileting, transferring, and/or continence) and/or the instrumental activities of daily living (IADL; i.e., housework, shopping, managing finances, taking medications as prescribed, and/or driving a vehicle) [3]. Due to this functional impairment in individuals with mild dementia, there may be concern about their ability to drive a vehicle.

Estimates of the prevalence of dementia demonstrate a wide range, wherein in individuals over the age of 65 years there is a prevalence of 6–10% whereas in individuals over the age of 85 years there is a prevalence of 30–50% [4]. In addition, studies have demonstrated that dementia is more common in industrialized countries for various reasons possibly including longer life spans and exposure to more environmental pollutants [4]. There are various types of dementia, the most common types being Alzheimer's disease (AD), dementia with Lewy bodies (DLB), and vascular dementia (VaD) [5]. Several medical conditions can also lead to the development of dementia, including Huntington's disease, multiple sclerosis, HIV, Parkinson's disease, Pick's disease, and progressive supranuclear palsy [6]. However, when examining the dementia population, AD and DLB are two of the most common types of dementia, accounting for over 60% of all dementia diagnoses [7].

AD and DLB are different in prevalence and cognitive profile. AD is the most commonly diagnosed form of dementia. The prevalence estimates change radically depending on the sample; for example, about 5% of people between the ages of 65 and 74 have AD, whereas nearly half the people over the age of 85 have AD. The average duration is approximately 10 years but includes a range of anywhere from 3 to 20 years [8]. AD is most commonly characterized by deficits in memory, attention, executive functioning, and language [5]. Even in mild AD, marked deficits in episodic memory are present; significant impairment across the attentional domain along with moderate deficits in language and executive functioning are also prevalent [5]. DLB is a neurodegenerative disease that may develop in old age, producing a combination of dementia, parkinsonism, and mental disturbances in the form of hallucinations [9]. The average age of onset of DLB is 67 years and the average duration of the illness is nine years [10]. According to autopsy reports, DLB accounts for 15–20% of all dementias [7]. In terms of cognitive deficits, individuals with DLB have significant visuospatial and perceptual deficits along with moderate attentional deficits [11]. Memory deficits are not marked in the early stages of the disease but as the disease progresses it also becomes impaired. Early presenting symptoms of DLB have not been studied extensively, especially in relation to mild AD [11].

There is an increased interest in examining the driving abilities of older adults diagnosed with dementia, especially those in early stages of the disease when they are most likely to be still driving actively (e.g., [12]). Certainly there are many benefits associated with driving an automobile, including better control of transportation timing, widespread accessibility of locations, access to employment and essential needs, increased social participation, and a sense of autonomy and independence. In fact, Carp [13] draws an important connection between mobility and quality of life, stating that “well-being depends on success in meeting life-maintenance and higher-order needs. Satisfaction of any need depends on congruence between the need and the resources for meeting it. Mobility is a key factor in determining congruence, because community services and facilities are irrelevant if they are inaccessible.” Thus, even though mobility is often measured in the number of trips an individual completes, the concept may be more related to the ability to access services and social interaction. Due to the link between quality of life and mobility through the use of a personal automobile there is interest in maintaining mobility in the mild dementia population so long as an individual can continue to drive safely.

Driving is considered to be a complex and dynamic task involving primarily cognitive (e.g., attention), perceptual (e.g., visual perception), and psychomotor processes (e.g., reaction time). Research has demonstrated that deficits in cognitive domains such as attention, global functioning, and visuospatial abilities are linked to impaired driving in driver's with mild dementia [14, 15]. However, a diagnosis of mild dementia does not necessarily signify the inability to drive safely or the necessity to revoke driving privileges, since individuals with a mild dementia diagnosis continue to drive for an average of 4 years following their diagnosis (e.g., [12]). Eby et al. [16], for example, used in-vehicle technology to explore the on-road driving behaviours of individuals with mild dementia and compared this behaviour to individuals without cognitive impairment. While the mild dementia group was found to significantly restrict their driving, they were found to drive as safely as the control group.

Our previous work using a driving simulator showed that drivers with mild dementia had significantly more errors and crashes during a standardized simulator assessment course [14, 15]. In addition, we found measures of global cognition, attention, and visuospatial processing were significantly related to simulator performance among individuals with mild dementia; however, the associations depended on dementia type. Numerous other studies have examined the relationship between neuropsychological test performance and indicators of driving ability among individuals with dementia; however, the results are mixed [17]. This may be attributed to the fact that driving is a complex task and that mild dementia patients are a rather heterogeneous population. Currently, there is no evidence to support using an individual cognitive or neuropsychological test to determine driving fitness [18]. Although examining the specific cognitive impairments associated with impaired driving is an important task, what has yet to be examined is the specific cognitive profile of licensed drivers with a mild dementia diagnosis.

Given that AD and DLB are the most common types of dementia, the purpose of this paper is to describe the cognitive profile of licensed drivers with mild AD and mild DLB. We hypothesized that, in comparison to healthy controls, mild AD drivers will exhibit impairments in attention, while mild DLB will demonstrate deficits in attention and visuospatial skills. The cognitive profiles of licensed drivers with mild AD and mild DLB will be compared to each other and to neurologically healthy older adult controls.

## 2. Method

### 2.1. Participants

There were three participant groups (*N* = 56) including a group of healthy older adult controls and two groups of individuals diagnosed with early stage dementia (DLB and AD). All participants were over the age of 65 years, were English speaking, and held a valid driver's license. The mean age of the control group was 77.00 years (SD = 5.86) with a range of 68 to 86 years, the mean years of education were 13.14 (SD = 3.18), and the group was comprised of 52.4% women and 47.6% men. The mean age of the early AD group was 78.50 years (SD = 7.22) with a range of 66 to 90 years, the mean years of education were 13.05 (SD = 3.94), and the group was comprised of 45% women and 55% men. Finally, the early DLB group had a mean age of 76.40 (SD = 6.59) with a range of 68 to 88 years, the mean years of education were 14.20 (SD = 4.55), and the group was comprised of 40% women and 60% men. Participants were matched for age and years of education.

A convenience sample of healthy older adult controls (*N* = 21) was obtained through advertisements in a community newspaper. These participants completed a 20-minute screening call in order to determine if they qualified to participate in this study. The exclusion criteria included any serious visual or hearing impairments left uncorrected, serious health problems, any medications that could alter cognitive abilities, any history of substance abuse, and any history of learning disabilities. For control participants, abnormal Mini-Mental State Exam (MMSE) scores (<25) was also grounds for exclusion; however, in practice no participants were excluded for this reason.

Participants diagnosed with mild dementia were a convenience sample recruited from a tertiary care facility in Ottawa. Participants who had a diagnosis of probable early DLB or AD at the memory clinic were contacted in order to determine their willingness to participate in the study. Participants were assessed for severity, using the Global Deterioration Rating Scale and only participants in the mild stages of dementia were included in this study (i.e., stages 3 and 4). The same exclusion criteria were used with the exception of medications, since the majority of participants with mild dementia were taking psychoactive medications, such as acetylcholinesterase inhibitors. Participants in the mild dementia were grouped in one of the two dementia groups depending on the diagnosis (i.e., AD or DLB).

In this study, all participants with dementia were diagnosed by the supervising neurologist at the memory clinic. All diagnoses of dementia were accomplished using a multimodal approach to diagnosis of dementia which greatly reduces diagnostic error [19]. Additionally, diagnosis of specific dementia group (AD and DLB) was accomplished using the current gold standards in diagnosis of dementia. DLB was diagnosed using the diagnostic criteria outlined by the first symposium on DLB, which has good predictive validity [20]. AD was diagnosed using the diagnostic criteria outlined by the National Institute of Neurological and Communicative Diseases and Stroke/Alzheimer's Disease and Related Disorders Association, which has excellent predictive validity (NINCDS-ADRDA) [21]. Using this method of diagnosis ensured that diagnostic groups had a good level of reliability.

### 2.2. Measures

#### 2.2.1. General Cognition

*Mini-Mental State Exam (MMSE)*. The MMSE is a brief cognitive screening tool that is based on a 30-point scale where 30 indicates the best performance. Typically, scores less than 24 indicate a cognitive abnormality or probable dementia. A score of 27 or greater is usually used to identify normal healthy adults. In the aging population, scores above 25 are used to identify normal healthy older adults, as such, only healthy controls with scores above and including 25 participated in this study. The MMSE has often been recommended as a screening tool for cognitive impairments in community dwelling older adults, as it has good sensitivity (80%) and high specificity (98%). The MMSE correlates (*r* = .79) with the cognitive and self-contained part of the Cambridge Examination for Mental Disorders of the Elderly (CAMCOG), the gold standard screening tool [22]. 

*Mattis Dementia Rating Scale (DRS-2 and DRS-2-Alternate)*. The DRS-2 is a measure of global neuropsychological functioning for older adults with suspected dementia; it assesses attention, memory, visuospatial construction, conceptualization, and initiation/perseveration [23–25]. Typically, higher functioning older adults can complete the battery in about 10 minutes, whereas participants with cognitive impairment may take approximately 45 minutes. The DRS-2 consists of 36 tasks and 32 stimuli, which yields five subscale scores and an assessment of the participant's overall level of cognitive functioning. The entire test was administered according to discontinue rules.

The DRS-2 has high sensitivity and specificity [26]. The reliability and validity properties of the DRS-2 are excellent. A test-retest reliability correlation coefficient was .97 with subscale correlation coefficients ranging from .61 to .94. The DRS was administered twice with a 1-week interval between administrations to a group of 30 participants diagnosed with dementia of the Alzheimer's type. A split-half reliability coefficient was .90, utilizing a sample of 25 participants aged 65 to 94 years who received diagnoses of either organic brain syndrome or senile dementia. A *t*-test indicated no significant differences between scores on the two halves. The alpha coefficients were calculated for four DRS subscales using a combined dementia sample. The alpha coefficients were attention (.95), initiation-perseveration (.87), conceptualization (.95), and memory (.75). The DRS-2 was compared with the Mini-Mental State Examination (MMSE), which displayed a significant correlation (*r* = .82) with the DRS-2 showing a greater sensitivity to change than the MMSE. In addition, correlations with the Wechsler Adult Intelligence Scale indicated a correlation of .75 between the WAIS full scale and the DRS-2 total score [24].

The DRS-2 and its alternate version were administered to all participants, with one version being administered in the first session and the second version in the second session. This was done in order to measure cognitive fluctuations between testing sessions, which are well-documented in individuals with DLB.

#### 2.2.2. Measures of Attention

*Test of Everyday Attention (TEA)*. The TEA is a measure of attention that specifically assesses various types of attention in an ecologically valid manner as the tests are related to everyday tasks [27]. This test has been normed on individuals from 18 to 80 years of age. There are eight subtests: (1) map search task, (2) elevator counting task, (3) elevator counting with distraction, (4) visual elevator task, (5) auditory elevator with reversal, (6) telephone search, (7) telephone search with dual task, and (8) lottery.

*Useful Field of View (UFOV)*. The UFOV is a measure of attention that is composed of three subtests: processing speed, divided attention, and selective attention [28]. Test performance on the UFOV has been related to performance on functional activities such as driving (e.g., [29]). UFOV scores among individuals with dementia have been shown to be more predictive of driving behaviour than dementia severity [30]. Test-retest reliability for the UFOV is high (*r* = .88; [31]).

#### 2.2.3. Measure of Perception

*Visual Object and Space Perception Test (VOSP)*. The VOSP is a measure of visuoperceptual and spatial abilities that assesses object and space perception [32]. The VOSP contains 8 subtests: shape detection, incomplete letters, silhouettes, object decision, dot counting, progressive silhouettes, position discrimination, number allocation, and cube analysis. Scoring for this test was completed for each individual subtest. In addition, a VOSP object perception composite score was calculated by adding the first four subtests and a VOSP space perception composite score was calculated by adding the last four subtests; these calculations were extracted from the user manual. A VOSP total score was also computed by summing all subtests.

#### 2.2.4. Measure of Language

*Boston Naming Test (BNT)*. The BNT is a test of word finding ability that consists of 60 large ink drawings that are presented in order of increasing difficulty [33]. Participants were asked to identify the picture correctly. In dementia research, it is common to provide cues to individuals who cannot name the picture [34]. At first a semantic cue is given (e.g., for a pelican, a semantic cue would be “it's a bird”), if the individual still does not respond a phonetic cue is provided (e.g., for a pelican, “pe…”). This test was normed on a population of 25 to 85 years of age. Normative data indicates that any score below 45 is abnormal. In this study, the total number of correctly identified drawings, whether the participant was cued or not, was used as the participant's score on this test (maximum score = 60).

### 2.3. Procedure

Potential control participants were contacted by telephone and were asked to complete a brief questionnaire to collect their demographic information. Patients with a dementia diagnosis were referred to the study by their neurologist. Patients who indicated interest in participating were contacted by telephone in order to verify that they were willing to participate and that they met the exclusion criteria. When a participant agreed to participate they were asked if they had time to answer a short questionnaire to collect their demographic information. In the event that they were unable to answer the demographic information on their own, a date was scheduled to have a phone interview with the participant and their primary caregiver. The recruitment of each participant was followed by a brief conversation with the supervising neurologists about the potential participant's ability to provide consent and to review the diagnosis and dementia stage.

All participants who met the inclusion criteria at the end of the demographic information questionnaire were asked for their availability. At this point the first of two testing sessions was scheduled with the participant. All participants began the first testing session by completing the consent form.

During the first testing session, participants with AD or DLB were asked questions pertaining to the Global Deterioration Rating Scale (GDS) in order to verify if they were in fact in the early stages of dementia (stages 3 and 4); participants whose disease was at other stages were excluded. Additionally, they completed the Mini-Mental State Exam (MMSE). The control participants were also asked to complete the MMSE.

Participants that met all the criteria participated in a first session of testing immediately following the screening. This session lasted approximately two and a half hours and participants were offered as many breaks as needed during the testing. All participants underwent a neuropsychological and computerized assessment including a test of general cognitive functioning (DRS-2), visuospatial/perceptual abilities (VOSP), word finding (BNT), attention (TEA), and processing speed (UFOV). Participants also underwent a simulated drive of approximately 20 minutes; the results of the simulated driving component are detailed elsewhere and a brief summary is included as part of the introduction of this article [14, 15]. All neuropsychological and computerized testing was completed according to the protocol specified by each measure. The neuropsychological and computerized testing was administered in the presence of the participant and the investigator only. Once the first session of testing was completed (i.e., consent signed, GDS completed for participants with AD and DLB, GDS depression, MMSE, DRS-2, the BNT, and the VOSP) another testing session was booked. During this second session, the DRS-2 was administered a second time, the TEA was completed, and the UFOV and the simulated driving task were administered. The DRS-2 and its alternate form were used and the order from sessions 1 to 2 was counterbalanced between participants. Both sessions lasted approximately 2.5 hours for a total of 5 hours of testing.

## 3. Results 

In order to compare the cognitive performance between the three groups, a series of between subjects ANOVAs were executed where group had three levels (i.e., AD, DLB, and control) and each of the neuropsychological measures was treated as dependent variables. Statistically significant omnibus ANOVAs were subsequently examined through Tukey's LSD test, a post hoc test that corrects for multiple comparisons. Descriptive statistics and the results of the omnibus tests are presented in Table 1.

### 3.1. Global Cognition

The analysis involving the MMSE and the DRS total indicated a statistically significant effect of group *F*(2,53) = 19.99 and *p* < .001. Post hoc analysis showed that, for both the MMSE and DRS measures, AD and DLB participants scored significantly poorer than healthy controls (see Table 2). There were no statistically significant differences between AD and DLB participants.

### 3.2. Attention

Measures of attention (i.e., UFOV and TEA) showed a statistically significant effect of group on all measures. When comparing dementia groups, the results showed DLB participants performed worse than AD participants on the TEA subtest 1 (i.e., map search 1 minute and 2 minutes) as well as the TEA subtest 2 (i.e., auditory elevator counting). Results of the post hoc analysis showed that UFOV-processing speed was significantly different between all three groups with DLB participants exhibiting the slowest scores and healthy controls showing the highest scores (see Table 3). UFOV-divided attention and UFOV-sustained attention showed a similar pattern of results where both AD and DLB participants scored significantly poorer than healthy controls. There were not statistically significant differences between AD and DLB participants in terms of divided attention and sustained attention.

### 3.3. Visuospatial and Perceptual Abilities

A statistically significant effect of group was noted for the majority of VOSP subtests as well as the VOSP object, space, and total composite scores. Through examining the post hoc contrasts, it was observed that AD and DLB participants performed poorer than controls based on the VOSP object composite scores (see Table 4). However, only the DLB group performed poorer than controls based on the VOSP space and total composite scores.

### 3.4. Language

Results from the analysis including the BNT as the dependent variable showed a statistically significant omnibus ANOVA, *F*(2,53) = 7.16 and *p* = .002. Multiple comparisons found that both dementia groups performed worse than healthy controls (see Table 5).

### 3.5. Cognitive Fluctuations

Cognitive fluctuations were assessed based on changes in participants' DRS scores over a one-week period. A statistically significant effect of group was noted when including the change in the DRS total score, *F*(2,53) = 3.24 and *p* = .047. Through inspection of the multiple comparison results, the data showed that only AD participants exhibited statistically significant cognitive fluctuations in comparison to healthy controls (*p* = .014; see Table 6).

## 4. Discussion 

The purpose of this manuscript was to present the cognitive profile of licensed drivers with mild AD and mild DLB and contrast their performance with group of healthy controls. We administered a number of relevant neuropsychological measures within the domains of general cognition, attention, visuospatial/perception, language, andcognitive fluctuations.

Our results indicated differences between healthy controls and demented participants on almost all neuropsychological measures. These striking differences are surprising given that all demented participants were in the mild stages of the disease (i.e., stages 3 and 4). Typically, this population is able to function autonomously and maintain their activities of daily living (ADLs). In light of these sweeping differences in cognitive function and considering that all individuals in this sample were licensed drivers, it is important for clinicians to consider that statistically significant differences between demented and healthy participants may not translate into functional impairments.

The results also showed that DLB drivers exhibited poorer attentional and visuospatial abilities in comparison, not only to controls, but also with reference to AD participants. Indeed, the clinical presentation for DLB includes visuospatial and attentional impairments whereas a hallmark for AD is memory impairment. Thus, these differences in neuropsychological test performance between dementia groups are consistent with the clinical and research literature [11].

The analysis presented here contributes to the body of knowledge used by clinicians and researchers who work with dementia populations. In particular, the norms we present were based on data collected from an educated sample of licensed drivers in an urban Canadian setting. When comparing these norms to individual patient performance, clinicians and health professionals should take into consideration these contextual factors.

An important inclusion criterion for this particular study was the possession of a valid driver's license and all participants reported being active drivers at the time of data collection. A limitation to this study was that we did not collect data regarding on-road driving behaviour. Such data might include self- or police-reported collisions, self-reported driving behaviour, or an on-road evaluation. The collection of these additional measures would allow for the determination of whether the differences in neuropsychological performance noted here correspond to indices of driving safety. A final limitation is the modest size of our sample. Future research should examine the value of the measures we administered in predicting driving safety among demented drivers using a large sample of participants.

Driving is multifactorial and complex behaviour that supports mobility and has been related to quality of life. Many healthcare professionals are tasked with making recommendations regarding fitness to drive and it is thus important for them to weigh both mobility needs and safety concerns when making this recommendation. Clinicians should triangulate multiple sources of information including subjective complaints, family reports, neuropsychological test performance, neurological assessment, and indicators of driving safety (i.e., on-road or simulator assessment). The norms presented in this paper may be useful to clinicians who are looking for comparative data when assessing patients. As previously noted there is currently no individual test to accurately predict driving safety. Research should continue to examine the predictive value of individual sources of information to support the mobility and safety needs of older adults with cognitive impairment.

## 5. Conclusion

Estimates suggest that AD and DLB are the most common forms of dementia among older adults. Driving is primary means of mobility among older adults and has been shown to contribute to quality of life. Researchers and clinicians suggest that individuals in the mild stages of cognitive impairment can often drive safely (e.g., Eby et al. [16]). The norms presented here suggest statistically significant differences between demented and healthy participants on almost all neuropsychological tests that were administered. However, in the absence of indices of driving behaviour, it is difficult to discern whether these significant differences result in meaningful impacts in terms of activities of daily living, including driving ability.

## Figures and Tables

**Table 1:** One-way ANOVA between controls (Ctrls), mild DLB, and mild AD on neuropsychological tests.

Neuropsych. tests	Mean AD	SD AD	Mean DLB	SD DLB	Mean Ctrls	SD Ctrls	df	*F*	*p*
MMSE	24.00	4.86	22.40	2.80	29.00	1.30	2, 53	19.99	<.001
DRS total	115.00	12.96	108.60	17.57	136.38	4.43	2, 53	26.78	<.001
Attention	35.15	2.06	33.00	4.31	36.29	.72	2, 53	7.19	.002
Initiat./persever.	27.20	7.43	27.47	5.59	34.91	3.25	2, 53	11.81	<.001
Construction	5.80	.52	4.00	1.25	6.00	.00	2, 53	39.12	<.001
Conceptualization	32.80	3.49	30.40	6.87	35.52	1.81	2, 53	6.48	.003
Memory	14.05	3.79	13.73	3.24	23.67	0.80	2, 53	76.53	<.001
BNT	43.00	12.30	46.6	9.74	53.62	3.26	2, 53	7.16	.002
VOSP	107.50	13.26	96.40	11.85	113.43	7.67	2, 53	10.44	<.001
VOSP1 (map search)	18.50	3.10	17.27	2.71	19.24	1.30	2, 53	2.83	.068
VOSP2 (elevator counting)	15.30	5.00	14.13	3.11	18.62	4.56	2, 53	5.24	.008
VOSP3 (elevator counting with distraction)	16.35	2.49	14.60	2.38	17.29	1.77	2, 53	6.47	.003
VOSP4 (visual elevator)	13.35	4.04	15.20	2.98	12.81	3.25	2, 53	2.16	.125
VOSP5 (elevator counting with reversal)	9.55	1.95	8.27	1.28	9.67	0.48	2, 53	5.26	.008
VOSP6 (telephone search)	17.85	3.34	16.93	4.43	19.14	1.11	2, 53	2.30	.110
VOSP7 (telephone search while counting)	9.45	3.30	6.13	3.38	8.62	1.91	2, 53	5.96	.005
VOSP8 (lottery)	7.15	2.78	3.87	3.66	8.05	2.73	2, 53	8.89	<.001
VOSP object (1–4)	63.50	7.13	61.20	4.35	67.95	5.82	2, 53	6.02	.004
VOSP space (5–8)	44.00	7.41	35.20	10.58	45.48	4.62	2, 53	8.95	<.001
TEA1: 1 minute (incomplete letters)	19.85	14.68	9.47	4.44	28.62	10.26	2, 53	13.17	<.001
TEA1: 2 minutes (incomplete letters)	30.35	19.71	14.93	9.16	52.19	11.87	2, 53	29.49	<.001
TEA2 (silhouettes)	6.75	.72	5.87	1.69	7.05	0.74	2, 53	5.38	.007
TEA3 (object decisions)	4.30	2.62	4.87	2.59	7.29	2.90	2, 53	6.86	.002
TEA4: raw score (progressive silhouettes)	3.05	3.25	2.73	3.15	6.91	1.95	2, 53	13.38	<.001
TEA5 (dot counting)	1.60	1.70	1.67	1.40	4.81	2.11	2, 53	20.54	<.001
TEA6 (position discrimination)	8.66	8.35	11.66	8.98	3.93	1.01	2, 53	5.93	.005
TEA7 (number location)	64.21	135.01	9.45	6.56	2.23	1.46	2, 53	3.45	.039
TEA8 (cube analysis)	3.75	3.04	4.00	3.00	7.95	1.69	2, 53	16.30	<.001
UFOV1 (processing speed)	143.15	142.79	272.07	120.32	24.91	16.74	2, 53	23.91	<.001
UFOV2 (divided attention)	406.10	130.90	432.20	135.17	152.52	116.45	2, 53	28.91	<.001
UFOV3 (selective attention)	452.40	90.86	490.20	37.96	304.76	115.13	2, 53	21.85	<.001
DRS differences	7.65	5.36	5.73	4.94	4.00	3.38	2, 53	3.24	.047
Attention	3.00	6.10	3.53	3.54	0.81	0.75	2, 53	2.35	.062
Initiat./persever.	5.10	4.19	2.93	3.63	2.38	3.35	2, 53	2.93	.062
Construction	.95	3.12	1.40	1.24	0.00	0.00	2, 53	2.42	.098
Conceptualization	4.85	7.53	2.80	2.27	2.10	1.84	2, 53	1.79	.177
Memory	2.95	5.71	2.47	1.99	1.10	0.70	2, 53	1.46	.242

**Table 2:** Post hoc group comparisons (i.e., control, DLB, and AD) on global measures of cognition.

Variable	Group	Comparison group	Mean difference	Std. error	*p*
MMSE	AD	Control	−5.00	1.04	<.001
		DLB	1.60	1.14	.167
	DLB	AD	−1.60	1.14	.167
		Control	−6.60	1.13	<.001

DRS1 total	AD	Control	−21.38	3.82	<.001
		DLB	6.40	4.17	.131
	DLB	AD	−6.40	4.17	.131
		Control	−27.78	4.13	<.001

Attention	AD	Control	−1.14	.80	.164
		DLB	2.15	.88	.018
	DLB	AD	−2.15	.88	.018
		Control	−3.29	.87	<.001

Initiation/perseveration	AD	Control	−7.71	1.77	<.001
		DLB	−.267	1.93	.891
	DLB	AD	.267	1.93	.891
		Control	−7.44	1.91	<.001

Construction	AD	Control	−.20	.22	.376
		DLB	1.80	.25	<.001
	DLB	AD	−1.80	.25	<.001
		Control	−2.00	.24	<.001

Conceptualization	AD	Control	−2.72	1.33	.045
		DLB	2.40	1.45	.104
	DLB	AD	−2.40	1.45	.104
		Control	−5.12	1.44	.001

Memory	AD	Control	−9.62	.89	<.001
		DLB	.32	.98	.747
	DLB	AD	−.32	.98	.747
		Control	−9.93	.97	<.001

**Table 3:** Post hoc group comparisons (i.e., control, DLB, and AD) on measures of attention.

Variable	Group	Comparison group	Mean difference	Std. error	*p*
TEA1min (map search)	AD	Control	−8.77	3.45	.014
		DLB	10.38	3.78	.008
	DLB	AD	−10.38	3.78	.008
		Control	−19.15	3.74	.000

TEA12min (map search)	AD	Control	−21.84	4.58	<.001
		DLB	15.42	5.00	.003
	DLB	AD	−15.42	5.00	.003
		Control	−37.26	4.95	<.001

TEA2 (elevator counting)	AD	Control	−.15	.31	.617
		DLB	.88	.34	.011
	DLB	AD	−.88	.34	.011
		Control	−1.04	.33	.003

TEA3 (elevator counting with distraction)	AD	Control	−2.99	.85	.001
		DLB	−.57	.93	.545
	DLB	AD	.57	.93	.545
		Control	−2.42	.92	.011

TEA4RAW (visual elevator)	AD	Control	−3.86	.88	<.001
		DLB	.32	.96	.742
	DLB	AD	−.32	.96	.742
		Control	−4.17	.95	<.001

TEA5 (elevator counting with reversal)	AD	Control	−3.21	.56	<.001
		DLB	−.07	.61	.914
	DLB	AD	.07	.61	.914
		Control	−3.14	.61	<.001

TEA6 (telephone search)	AD	Control	4.73	2.13	.031
		DLB	−3.00	2.33	.203
	DLB	AD	3.00	2.33	.203
		Control	7.74	2.31	.001

TEA7 (telephone search while counting)	AD	Control	61.98	25.28	.018
		DLB	54.76	27.64	.053
	Control	AD	−61.98	25.28	.018
		DLB	−7.22	27.35	.793
	DLB	AD	−54.76	27.64	.053
		Control	7.22	27.35	.793

TEA8 (lottery)	AD	Control	−4.20	.81	<.001
		DLB	−.25	.89	.780
	DLB	AD	.25	.89	.780
		Control	−3.95	.88	.000

UFOV1 (processing speed)	AD	Control	118.25	33.12	.001
		DLB	−128.92	36.21	.001
	DLB	AD	128.92	36.21	.001
		Control	247.16	35.84	<.001

UFOV2 (divided attention)	AD	Control	253.58	39.63	<.001
		DLB	−26.10	43.32	.549
	DLB	AD	26.10	43.32	.549
		Control	279.68	42.88	<.001

UFOV3 (selective attention)	AD	Control	147.64	28.54	<.001
		DLB	−37.80	31.20	.231
	DLB	AD	37.80	31.20	.231
		Control	185.44	30.88	<.001

**Table 4:** Post hoc group comparisons (i.e., control, DLB, and AD) on measures of visuospatial/perceptual abilities.

Variable	Group	Comparison group	Mean difference	Std. error	*p*
VOSP total	AD	Control	−5.93	3.46	.092
		DLB	11.10	3.78	.005
	DLB	AD	−11.10	3.78	.005
		Control	−17.03	3.74	<.001

VOSP1 (incomplete letters)	AD	Control	−.74	.77	.341
		DLB	1.23	.84	.147
	DLB	AD	−1.23	.84	.147
		Control	−1.97	.83	.021

VOSP2 (silhouettes)	AD	Control	−3.32	1.38	.019
		DLB	1.17	1.50	.441
	DLB	AD	−1.17	1.50	.441
		Control	−4.49	1.49	.004

VOSP3 (object decisions)	AD	Control	−.94	.69	.182
		DLB	1.75	.76	.025
	DLB	AD	−1.75	.76	.025
		Control	−2.69	.75	.001

VOSP4 (progressive silhouettes)	AD	Control	.54	1.09	.622
		DLB	−1.85	1.19	.127
	DLB	AD	1.85	1.19	.127
		Control	2.39	1.18	.048

VOSP5 (dot counting)	AD	Control	−.12	.43	.787
		DLB	1.28	.47	.009
	DLB	AD	−1.28	.47	.009
		Control	−1.40	.47	.004

VOSP6 (position discrimination)	AD	Control	−1.29	.97	.189
		DLB	.92	1.06	.392
	DLB	AD	−.92	1.06	.392
		Control	−2.21	1.05	.040

VOSP7 (number location)	AD	Control	.83	.90	.360
		DLB	3.32	.98	.001
	DLB	AD	−3.32	.98	.001
		Control	−2.49	.97	.014

VOSP8 (cube analysis)	AD	Control	−.90	.94	.346
		DLB	3.28	1.03	.002
	DLB	AD	−3.28	1.03	.002
		Control	−4.18	1.02	<.001

VOSP object (1–4)	AD	Control	−4.45	1.88	.021
		DLB	2.30	2.05	.267
	DLB	AD	−2.30	2.05	.267
		Control	−6.75	2.03	.002

VOSP space (5–8)	AD	Control	−1.48	2.36	.535
		DLB	8.80	2.56	.001
	DLB	AD	−8.80	2.56	.001
		Control	−10.28	2.56	<.001

**Table 5:** Post hoc group comparisons (i.e., control, DLB, and AD) on measures of language.

Group	Comparison group	Mean difference	Std. error	*p*
AD	Control	−10.62	2.85	<.001
	DLB	−3.60	3.12	.253

DLB	AD	3.60	3.12	.253
	Control	−7.02	3.09	.027

**Table 6:** Post hoc group comparisons (i.e., control, DLB, and AD) on measures of cognitive fluctuations.

Dependent variable	Group	Comparison group	Mean difference	Std. error	*p*
DRS total absolute differences	AD	Control	3.65	1.43	.014
		DLB	1.92	1.57	.227
	DLB	AD	−1.92	1.57	.227
		Control	1.73	1.55	.269
